# Synthesis and biological activities of two camptothecin derivatives against *Spodoptera exigua*

**DOI:** 10.1038/s41598-019-54596-y

**Published:** 2019-12-02

**Authors:** Fulai Yang, Liping Wang, Lan Zhang, Yanning Zhang, Liangang Mao, Hongyun Jiang

**Affiliations:** 1 State Key Laboratory for Biology of Plant Disease and Insect Pests, Institute of Plant Protection Chinese Academy of Agricultural Sciences, Beijing, 100193 China; 2 Ministry of Agriculture and Rural Affairs of People’s Republic of China, Beijing, 100193 China

**Keywords:** Chemical biology, Chemical biology, Natural product synthesis, Natural product synthesis

## Abstract

Camptothecin (CPT), a natural alkaloid isolated from *Camptotheca acuminata* Decne, is found to show potential insecticidal activities with unique action mechanisms by targeting at DNA-topoisomease I (Top1) complex and inducing cell apoptosis. To improve the efficacy against insect pests, two camptothecin (CPT) derivatives were synthesized through introducing two functional groups, 2-nitroaminoimidazoline and 1-chloro-2-isocyanatoethane by esterification reaction. The insecticidal activities of these two derivatives were evaluated at contact toxicity, cytotoxicity and topoisomerase I (Top1) inhibitory activities comparing with CPT and hydroxyl-camptothecin (HCPT). Results showed that compound a, synthesized by introducing 2-nitroaminoimidazoline to CPT, apparently increased contact toxicity to the third larvae of beet armyworm, *Spodoptera exigua*, and cytotoxicity to IOZCAS-Spex-II cells isolated from *S. exigua*. However, the inhibition on DNA relaxation activity of Top1 was reduced to less than 5 percentage even at high concentrations (50 and 100 μM). For introducing 1-chloro-2-isocyanatoethane to HCPT, the contact toxicity, cytotoxicity and Top1 inhibitory activity of synthesized compound b were increased significantly compared to CPT and HCPT. These results suggested that both synthesized compounds possessed high efficacy against *S. exigua* by targeting at Top1 (compound b) or novel mechanism of action (compound a).

## Introduction

**Figure 1:**
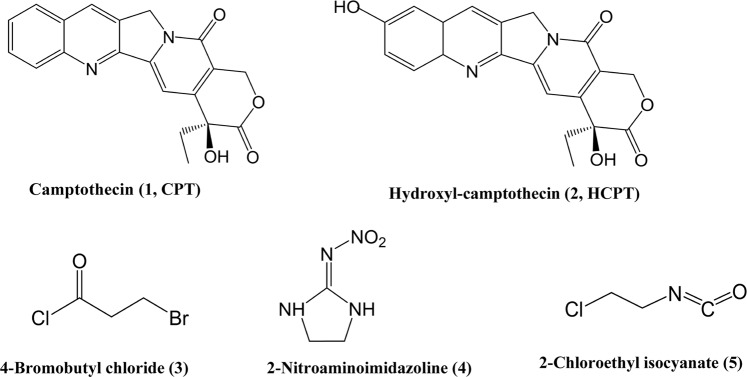
Structures of camptothecin (CPT, 1), hydroxy-camptothecin (HCPT, 2), 4-brommobutyl chloride (3), 2-nitroaminoimidazoline (4), 2-chloroethyl isocyanate (5).

Plant secondary metabolites have a long history as a source of, and inspiration for, novel insect control agent. Examples include pyrethrum, nicotine, rotenone, which were used directly for pest control and/or as the lead compounds to develop insecticides. Camptothecin (CPT, 1, Fig. 1) is a naturally occurring alkaloid believed to serve as a defense against insect herbivores^1,2^. In China, the farmer had applied historically the crude extract of *C. acuminata* to control insect pests^3^. Recently, much interest has been focused on CPT due to its bioactivities to some important pest insects and unique action mechanisms by targeting at DNA and topoisomerase I (Top1) complex and inducing cell apoptosis^4–6^.

Several studies have demonstrated that CPT shows toxic effects on fruit flies (*Drosophila melanogaster* Meigen)^7^, house flies (*Musca domestica* Linnaeus)^2^, and several important agricultural pest species including *Spodoptera exigua* Hübner^3^, *Nilaparvata lugens* Stål, *Brevicoryne brassicae* Linnaeus, and *Chilo suppressalis* Walker^8^. Interestingly, Sun *et al*. evaluated CPT synergistic effects on *Bacillus thuringiensis* (*Bt*) *var. kurstaki* and nucleopolyhedroviruses against *Trichoplusia* ni (Hübner) and *S. exigua*^9^. Results showed that CPT enhanced significantly the toxicity of *Bt var. kurstaki* to *S. exigua* and *T. ni*, and the infectivity of *Autographa californica* (Speyer) multinucleocapsid nucleopolyhedrovirus (AcMNPV) and *S. exigua* nucleopolyhedrovirus (SeMNPV). CPT and its derivatives, hydroxylcamptothecin (HCPT, 2, Fig. 1) could induce apoptosis in insect cell lines, such as IOZCAS-Spex-II (established from *S. exigua*)^10^, BmN-SWU1 (established from *Bombyx mori* Linnaeus)^11,12^, SL-1 (established from *Spodoptera litura* Fabricus)^13^, Sf9 and Sf21 (isolated from *Spodoptera frugiperda* Smith)^10,14^. In BmN-SWU1 and IOZCAS-Spex-II, it was documented that CPT and/or HCPT initiated the apoptosis through the intrinsic mitochondrial pathway^12,15^. Furthermore, CPT and HCPT showed inhibitory effects on DNA relaxation activities of Top1 extracted from IOZCAS-Spex-II cells, and reduced the steady accumulation of Top1 protein in IOZCAS-Spex-II^16^.

However, CPT has obvious shortcomings and drawbacks including low water solubility and poor cuticular penetrability^17^. In addition, the lactone ring of CPT is unstable which makes it easy transform to inactive carboxylate compound. In order to improve the physical-chemical property and biological activity of CPT, chemistry efforts developed several methods to synthesize CPT derivatives^18,19^. It has been documented to be practicable to introduce a suitable functional structure to CPT for improving efficacy. Liu *et al*. synthesized a series of novel CPT derivatives through substituting at C-20-hydroxyl of CPT with nitroxides to improve their bioactivities against *Mythimna separate* Walker to a certain degree and solubility in most organic solvents^20^. Their group also incorporated three functional fragments (ureas, thioureas, and acylthioureas) into CPT at C-7 position and synthesized three series of novel CPT derivatives. Based on the observed bioactivities, all synthesized compounds showed more potent that CPT against *Tetranychus Cinnabarinus* Boisduval, *Brevicoryne brassicae* Linnaeus, and *Bursaphelenchus xylophilus* Steiner et Buhrer^21^. Our previous studies showed that introduction of *t*-Boc amino acid and chrysanthemic acid to CPT could improve contact activities, cytotoxicity of most derivatives against *S. exigua*^22,23^.

In this paper, we aimed to improve CPT efficacy by introducing two functional groups, 2-nitroaminoimidazoline and 1-chloro-2-isocyanatoethane to CPT. The contact toxicities of target compounds against third-instar larvae of *S. exigua* was tested, and the cytotoxicity was detected by 3-(4,5-dimethylthiazol-2-yl)-2,5-diphenyltetrazolium bromide (MTT) assay with IOZCA-Spex-II cell lines. Meanwhile, we evaluated the inhibition effect of these two target derivatives on DNA relaxation activity of Top1.

## Results

### Contact toxicity

The contact toxicity of target compounds a and b was tested against the third-instar larvae of *S. exigua* compared to CPT and HCPT. As shown in Table 1, the LD_50_ values were 8.22, 4.63 and 3.24 μg/larva for compound a, and 10.8, 10.3 and 5.68 μg/larva at 24, 48 and 72 h, respectively. However, the values of LD_50_ were not detectable at the tested concentrations (0.625, 1.25, 2.5, 5 and 10 mg/ml) for CPT and HCPT, except for HCPT at 72 h (LD_50_, 10.7 μg/larva). The contact toxicity of compounds a and b against the third instar larvae of *S. exigua* was increased significantly. Especially, the relative speed of toxic effect was increased with significantly higher corrected mortality 58.3% and 51.7% for compounds a and b than 1.70% and 20.0% for CPT and HCPT at 24 h, respectively (data not shown). These results showed that the bioactivity was improved by introducing 2-nitroaminoimidazoline and 1-chloro-2-isocyanatoethane to CPT, respectively.

**Table 1:** Contact toxicity of target compounds a and b compared to CPT and HCPT against the third-instar larvae *S. exigua*.

Compounds	24 h					48 h					72 h				
	LD_50_ (95%FL) μg/larvae	Slope ± SE	*df*	F	*P*	LD_50_ (95%FL) μg/larvae	Slope ± SE	*df*	F	*P*	LD_50_ (95%FL) μg/larvae	Slope ± SE	*df*	F	*P*
CPT	>10	—	—	—	—	>10	—	—	—	—	>10	—	—	—	—
HCPT	>10	—	—	—	—	>10	—	—	—	—	10.7 (4.63–24.8)	2.19 ± 0.75	4	8.40	0.062
a	8.22 (6.06–11.2)	1.08 ± 1.15	4	50.2	0.006	4.63 (3.69–5.81)	1.06 ± 0.10	4	72.1	0.003	3.24 (2.39–4.42)	0.91 ± 0.13	4	4.42	0.006
b	10.8 (3.39–34.4)	3.51 ± 1/66	4	4.45	0.125	10.3 (3.08–34.2)	3.48 ± 1.75	4	3.93	0.141	5.68 (3.81–8.46)	1.61 ± 0.33	4	23.7	0.016

LD_50_: median lethal dose,

FL: fiducial limit,

SE: standard error,

df: degree of freedom.

Data are shown as means ± SD. Means followed by different letters within the same column are significantly different (*P* < 0.05).

### Cytotoxicity

**Figure 2:**
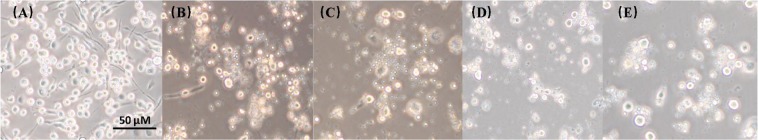
Morphology observation of IOZCAS-Spex-II cells treated with 10 μM CPT (**B**), HCPT (**C**), a (**D**) and b (**E**) for 72 h. 0.1% DMSO was used as a control (**A**). The scale bar is 50 μm.

**Figure 3:**
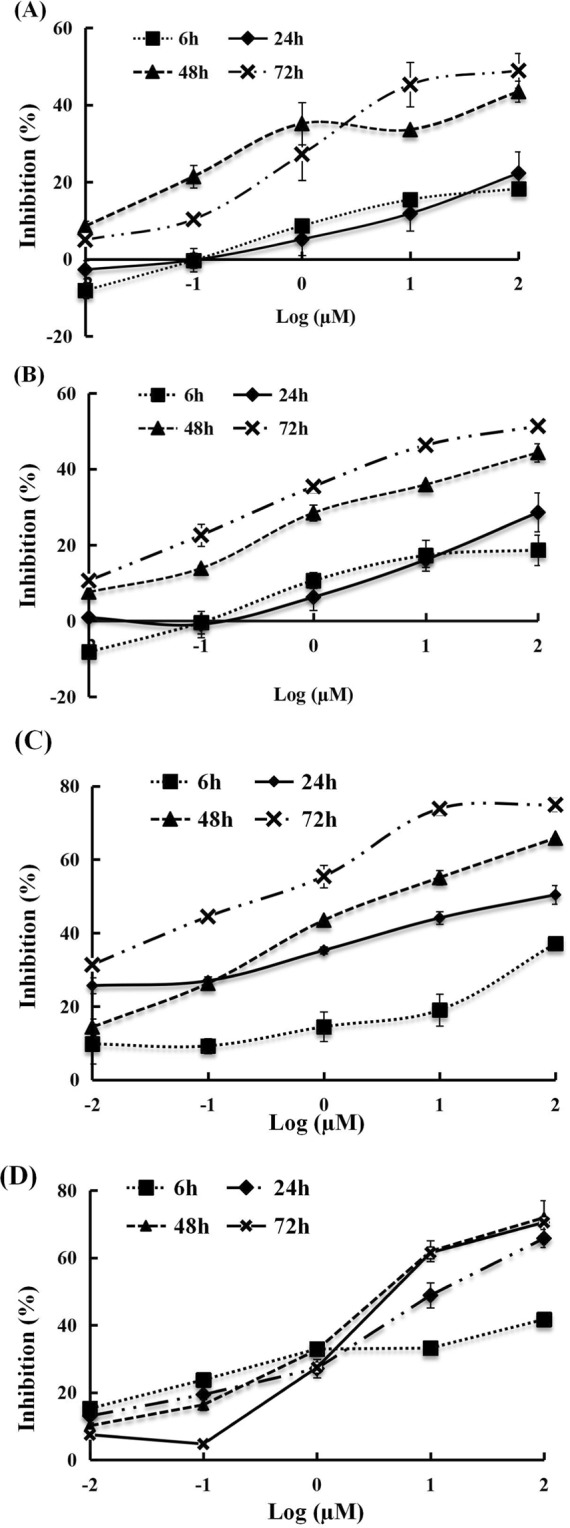
Inhibitory effects of CPT and its derivatives against *S. exigua* cell line IOZCAS-Spex-II. Inhibition (%) = (OD490 of the 0.1% DMSO-treated cells − OD490 of CPT or its analogues-treated cells)/OD of 0.1% DMSO-treated cells × 100%. Values are shown as mean ± SEM. (**A**) CPT; (**B**) HCPT; (**C**) Compound a; **(D**) Compound b.

To investigate whether the compounds (a, b) have the potent toxicity against the *S. exigua* cell line IOZCAS-Spex-II, cells were incubated with a series of dilutions (0.01–100 μM) of compounds at different times (6, 12, 48 and 72 h). As shown in Fig. 2A, cells treated with 0.1% DMSO in the control group were normal with long dendrites and axons, indicating good growth. After treated with 10 μM compounds for 72 h, typical apoptotic morphology (apoptotic body) was observed in CPT and HCPT treated groups, but for compounds a and b, cells showed damaged significantly. As shown in Fig. 3, compounds (a, b) exhibited cytotoxic effects on the *S. exigua* cell line IOZCAS-Spex-II in a time-and-dose-dependent manner. Compound a showed the highest cytotoxic effect proved by EC_50_ values 5.19 μM at 48 h and 0.29 μM at 72 h in Table 2. Compound b showed higher cytotoxicity with EC_50_ values 5.36 μM at 48 h and 9.43 μM at 72 h. Concerned synthetically results of contact bioassay and cytotoxicity assay, introducing groups of 2-nitroaminoimidazoline and 1-chloro-2-isocyanatoethane to CPT and HCPT improved bioactivities of CPT and HCPT against *S. exigua*.

**Table 2:** Cytotoxicity of target compounds a and b comparing with CPT and HCPT to the *S. exigua* cell line IOZCAS-Spex-II.

Compounds	48 h					72 h				
	EC_50_ (95%CL) μM	Slope ± SE	*df*	F	*P*	EC_50_ (95%FL) μM	Slope ± SE	*df*	F	*P*
CPT	>100	—	—	—	—	>100	—	—	—	—
HCPT	>100	—	—	—	—	32.4(9.92–105)	0.44 ± 0.05	3	62.2	0.0042
a	5.19 (3.22–8.40)	0.37 ± 0.02	3	222	0.0007	0.29(0.13–0.66)	0.30 ± 0.04	3	69.3	0.0036
b	5.36 (2.81–10.2)	0.49 ± 0.04	3	124	0.0015	9.43(1.94–45.8)	0.59 ± 0.12	4	24.0	0.0162

EC_50_: concentration for 50% of maximal effect,

FL: fiducial limit,

SE: standard error,

df: degree of freedom.

### Inhibition on the relaxation activity of Top1 in *S. exigua*

**Figure 4:**
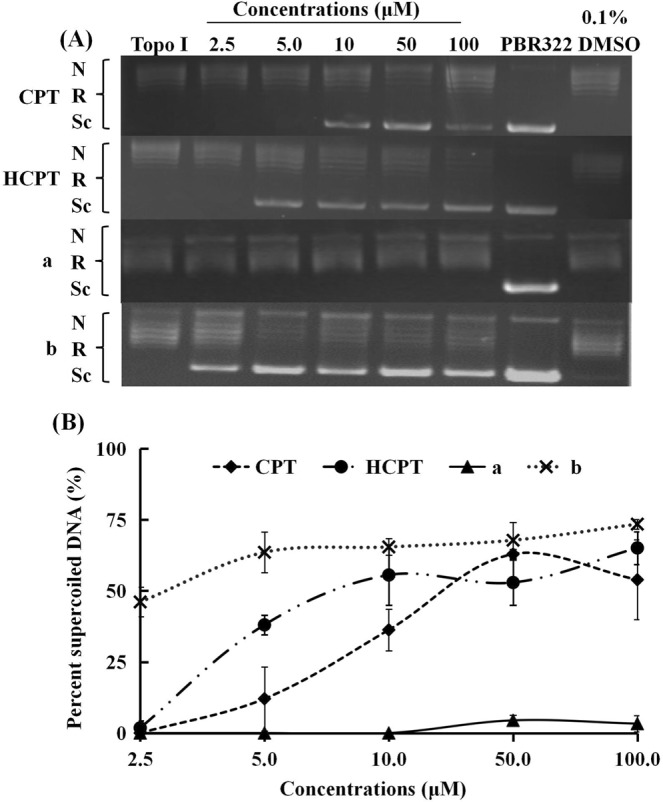
Inhibition of CPT and its derivatives on the DNA relaxation activity of *S. exigua* Top1. N, nicked DNA; R, relaxed DNA; Sc, supercoiled DNA; Top1, Top1 + pBR322 + 0.10% DMSO; pBR322, pBR322 + 0.10% DMSO. All the experiments were performed three times and one set of representative pictures were shown. Full-length gels are presented in Supplementary Fig. 1.

Top1 inhibitory activity of target compounds was assessed by using Top1 mediated relaxation assay. In this test, target compound b exhibited the maximum inhibitory effect (46.1%) even at lowest concentration 2.5 μM compared to all the other tested compounds in Fig. 4. The inhibition rate of compound b on Top1 relaxation activity reached to 73.5% when concentration reached to 100 μM. These data suggested that introducing 1-chloro-2-isocyanatoethane to 10 hydroxyl-position of HCPT improved significantly the inhibitory activity on Top1. However, target compound a showed almost no inhibitory activity on Top1 even at higher concentrations 50 and 100 μM. Together results of contact bioassay and cytotoxicity assay, it showed that substituting at C-20-hydroxyl of CPT could improve CPTs’ efficacy independent of Top1 inhibition.

## Discussion

CPT has been discovered from plant extracts since 60 years ago^24^. Its bioactivities against insects have been documented and suggested the potential in insect pest management. However, CPT’s drawbacks in both physical-chemical property limited its application. In order to improve CPT’s efficacy against insect pests, two compounds (a, b) were synthesized by condensation of CPT and HCPT respectively with functional groups 2-nitroiminoimidazolidine and 1-chloro-2-isocyanatoethane. Bioactivities of these two compounds were evaluated by contact toxicity assay, cytotoxicity test and Top1 inhibitory test compared to CPT and HCPT.

Compound b, synthesized by introducing 1-chloro-2-isocyanatoethane, exhibited higher contact toxicity and cytotoxicity against *S. exigua*, and the maximum inhibitory effect on Top1 relaxation activity among all tested compounds. It indicated compound b possessed potential to be developed as a pesticide against *S. exigua* by targeting at Top1. Compound a, synthesized by introducing 2-nitroiminoimidazolidine to 20-position on CPT, showed also higher contact toxicity and cytotoxicity against *S. exigua* than CPT an HCPT. However, the inhibition on Top1 relaxation activity of compound a was decreased to less than 5% even at concentrations 50 and 100 μM. In our previous study, introduction of *t*-Boc amino acids to 20-position on CPT improved contact assay and cytotoxicity of most derivatives toward *S. exigua* but reduced inhibitory effect on relaxation activity of *S. exigua* Top1^22^. DNA Top1 is the key enzyme that modulates the topological state of DNA through the breaking and rejoining of DNA strands. CPT has been shown to bind reversibly to the covalent intermediate DNA-Top1 and stabilize the cleavable complex, which collides with the progressing replication fork producing lethal double-stranded DNA breaks^25^. It has been demonstrated that DNA double strand breaks are responsible for the S-phase-specific cytotoxicity of CPT^26^. Our previously studies also confirmed that CPT and its derivatives can induce DNA fragment and cell apoptosis in *S. exigua*^15,16^. Meanwhile, DNA breaks induced by CPT and HCPT played an essential role in ROS over-generation which can lead to apoptosis^27^. Interestingly, some synthesized CPT analogues as drug for cancer treatment can improve efficacy independent of Top1 inhibition with novel mechanism of action by targeting important cancer survival-associated oncogenic proteins and/or by bypassing various treatment-resistant mechanisms^28,29^. Additionally, the authors evidence that CPT analogues exhibited high efficacy and independently of Top1 inhibition can exert low toxicity to the host because inhibition of Top1 activity may mainly be involved in toxicity. Therefore, novel CPT analogues with low toxicity due to not targeted at Top1 was proposed an important research area with great potential to make a breakthrough for development of the next generation of CPT derivatives for cancer treatment^27,30^. In this study, compound a possessed higher efficacy against *S. exigua* but lower inhibitory effects on Top1 activity, which indicated that this CPT analogue may possess lower Top1 toxicity to non-target organisms compared with, compound b, CPT and/or HCPT. It needed further studies and proposed an orientation in the development for CPT derivatives.

In summary, two kinds of functional group (2-nitroaminoimidazoline and 1-chloro-2-isocyanatoethane) were introduced to CPT, and then the contact toxicity, cytotoxicity and Top1 inhibitory activity were evaluated. Results showed both synthesized compounds possessed high efficacy against *S. exigua* by targeting at Top1 (compound b) or novel mechanism of action (compound a).

## Materials and Methods

### Chemistry general information

CPT (99.36%) and HCPT (99.45%, HCPT, 2; Fig. 1) were supplied by Knowshine Pharmachemicals Inc, Shanghai, China. N, N-Dimethylformamide (DMF), triethylamine and tetrahydrofuran (THF) were purchased from GL Biochem Co., Shanghai, China. 4-Bromobutyl chloride, 2-nitroaminoimidazoline, 2-chloroethyl isocyanate, 4-dimethylaminopyridine (DMAP) and diisopropylcarbinol (DIPC) were purchased from Sigma Aldrich Co., China. All the other reagents and solvents were commercial products of analytical grade and used directly without additional purification.

### Insect and cell line

A laboratory population of *S. exigua* reared at 25 ± 2 °C under a controlled 16:8 h light: dark photoperiod without exposure to any insecticides for more than ten years, was used in the bioassay. Cell line, IOZCAS-Spex-II was used for cytotoxicity assays, which was established form the fat body of *S. exigua* and offered by Prof. Qilian Qing of the Institute of Zoology, Chinese Academy of Sciences, Beijing, China. The cells were cultured in Grace’s insect culture medium supplemented with 10% fetal bovine serum in T25 cm^2^ tissue culture flasks (Corning, USA) at 27 °C. The culture was sub-cultured every 6 days.

### Synthesis of two novel CPT derivatives

**Figure 5:**
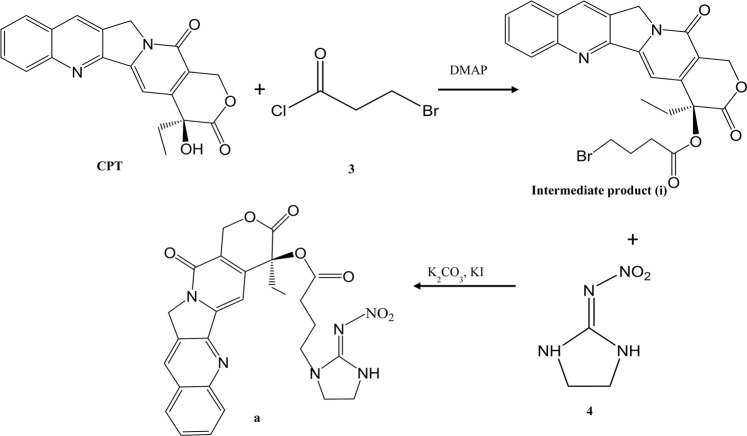
Synthesis of target compound a.

**Figure 6:**
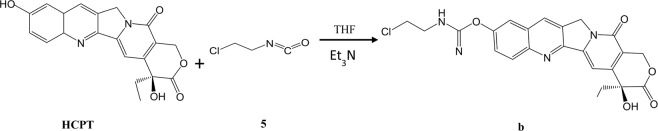
Synthesis of target compound b.

Two novel CPT derivatives were designed and synthesized by esterification with corresponding functional groups as shown in Figs. 5, 6.

#### Synthesis of target compound a

Under the protection of nitrogen, a solution of CPT (348 mg, 1 mM) and 4-bromobutyl chloride (514 mg, 3 mM) in dichloromethane (10 ml) were mixed with DMAP (122 mg, 1 mM). The reaction mixture was incubated at 30 °C for 6 h. After concentration and purification by Prep-HPLC, the intermediate (i) was obtained. Subsequently, the intermediated compound (i, 400 mg) and 2-nitroaminoimidazoline (390 mg, 3 mM) were dissolved in DMF (20 ml) and incubated at 30 °C for 6 h under nitrogen. The reaction mixture was concentrated and purified with Prep-HPLC to give the title compound a as 45 mg, white solid.

#### Synthesis of target compound b

To a stirred mixture of 2-chloroethyl isocyanate (633 mg, 6 mM) and HCPT (365 mg, 1 mM) in TFH (120 ml), triethylamine (1 ml) was added at room temperature, and then reacted for 24 h. The crude product was concentrated and then purified with Prep-HPLC, which resulted in the desired compound b (160 mg, light yellow solid).

#### General experimental procedures

In order to monitor the chemistry synthesis reaction, the analytical thin-layer chromatography (TLC) was conducted on the pre-coated plates (silica gel GF254).and the resulted spots were visualized with UV light. Melting points (mp) were determined on a X-4 microscopic melting point apparatus (Yu Hua, China) and were uncorrected. All synthesized title compounds were identified by high resolution mass spectrometry (HRMS) analyses on a Bruker Apex IV FTMS instrument (Bruker Company, Boston, MA, USA). The 1 H NMR spectra was recorded at 400 MHz on a Bruker AM-400 spectrometer (Bruker Company, Boston, MA, USA) in DMSO-d6 or CDCl_3_. Chemical shifts (δ) were expressed in part per million (ppm) with TMS as an internal standard.

#### Target compound a

Yeild: 52.9%; purity: 96.8%. MS (ESI): m/z (M + H)^+^ 547.2. ^1^H NMR (400 MHz, CHCl_3_-*d*) δ 8.42 (s, 1 H), 8.26 (d, *J = *8.53 Hz, 1 H), 8.17 (br s, 1 H), 7.96 (d, *J = *8.16 Hz, 1 H), 7.83–7.89 (m, 1 H), 7.67–7.73 (m, 1 H), 7.23 (s, 1 H), 5.70 (d, *J = *17.19 Hz, 1 H), 5.42 (d, *J = *17.19 Hz, 1 H), 5.32 (s, 2 H), 3.61–3.81 (m, 4 H), 3.45 (t, *J = *7.09 Hz, 2 H), 2.56–2.69 (m, 2 H), 2.24–2.33 (m, 1 H), 2.13–2.22 (m, 1 H), 1.93–2.00 (m, 2 H), 1.01 (t, *J = *7.47 Hz, 3 H) ppm.

#### Target compound b

Yeild, 44.3%; purity: 93.5%. MS (ESI): m/z (M + H)^+^ 470.0. ^1^H NMR (400 MHz, DMSO-d6) δ = 8.67 (s, 1 H), 8.28 (t, *J* = 5.75 Hz, 1 H), 8.18 (d, *J = *9.17 Hz, 1 H), 7.91 (d, *J* = 2.57 Hz, 1 H), 7.66 (dd, *J = *9.11, 2.51 Hz, 1 H), 7.34 (s, 1 H), 6.55 (s, 1 H), 5.44 (s, 2 H), 5.29 (s, 2 H), 3.74 (t, *J = *6.05 Hz, 2 H), 3.47 (q, *J = *5.95 Hz, 2 H), 1.88 (m, 2 H), 0.89 (t, *J = *7.34 Hz, 3 H) ppm.

### Contact toxicity

According to Huang *et al*.^31^, the bioassays were conducted with topical application method to assess the contact toxicity of target compounds towards the third-instar larvae of *S. exigua* comparing with CPT and HCPT. All test compounds were prepared in acetone at the concentrations of 0.625, 1.25, 2.5, 5 and 10 mg/ml. Total 1.0 μL solution of each test compounds was applied topically to the dorsal thorax of the third-instar larvae of *S. exigua* using a Gilson Pipetman Concept (EDP3-plus 1–10 μL). Ten larvae were used for each treatment. After treatment, all larvae were transferred to culture boxes and fed with fresh cabbage leaves in an incubator under conditions 25 ± 2 °C, 70% RH, and a photoperiod of 16:8 h (light: dark). The control was conducted simultaneously with acetone under the same conditions. All tests were repeated tree times. The larval mortality was observed at 24, 48 and 72 h after treatment.

### Cytotoxicity

Cell morphology was observed by using an inverted phase contrast microscope (Olympus IX51, OLYMPUS OPTICAL CO., LTD). The cytotoxicity of synthesized compounds against insect cells was detected with a Cell Titer 96 Aqueous One Solution Cell Proliferation Assay kit comparing with CPT and HCPT. According to the manufacturer’s instruction, IOZCAS-Spex-II cells in logarithmic phase were harvested and diluted to a density of 10^4^ cells/ ml. After that, cells were plated in 96-well plates (160 μL/well) and cultured for 12 h. Subsequently, total 10 μL tested compounds were added to each well at different concentrations (0.01–100 μM) for 6, 24, 48 and72 h. All tests were repeated tree times.Total 30 μL Cell Titer 96 One Solution Reagent was added to each well and incubated to the color changes for 2 h at 27 °C. The absorbance was read at 490 nm using a TMF200 microplate reader (Tecan, USA). EC_50_ values of each treatment were calculated by regression curve fitting by using DPS software analysis.

### Inhibition on the relaxation activity of Top1 in *S. exigua*

The inhibitory effects of all compounds on the activity of Top1 were detected byTop1 Top1 mediated DNA relaxation assay. Briefly, native Top1 was isolated from insect cells, IOZCAS-Spex-II, according to the method described in the previous publication^16^. Total 20 μL reaction mixture consisted of 1U of natural Top1, 0.5 μg supercoild pBR322 DNA, and 1 × reaction buffer (pH 7.5, 10 mM Tris-HCl, 1 mM DTT, 1 mM EDTA, 0.1 mg/ml BSA) was incubated with various concentrations of test compounds (from 0 to 100 μM) at 27 °C for 30 min. The reaction was terminated by the addition of a final 12.5 µg/µL proteinase K and 0.5% SDS. The products were analyzed by 1% gel electrophoresis and DNA bands were visualized after ethidium bromide staining on a UV light transilluminator and quantified with Quantity one analysis software (Gel Doc XR, Bio-Rad, USA). The inhibitory effects (%) of the tested compounds on Top1 relaxation activity was calculated as the percentage of supercoiled DNA over total pBR322 DNA.

### Statistical analysis

Statistical analyses were conducted with Ducan’s test using DPS software. All results were considered statistically significant (*P* < 0.05).

## Supplementary information

Supplementary Dataset 1
