# Supplementary material for: Synthesis and biological activities of two camptothecin derivatives against Spodoptera exigua
Source: Sci Rep. 2019 Dec 2;9:18067. doi: 10.1038/s41598-019-54596-y (PMC6889156; doi:10.1038/s41598-019-54596-y)
Supplement: Supplementary file 1 — Supplementary Dataset 1 [file 41598_2019_54596_MOESM1_ESM.pdf]

Title page

Title:

Synthesis and Biological activities of two camptothecin derivatives against *Spodoptera exigua*

Author names and affiliations:

Fulai Yang, Liping Wang, Lan Zhang*, Yanning Zhang, Liangang Mao & Hongyun Jiang*

State Key Laboratory for Biology of Plant Disease and Insect Pests, Institute of Plant Protection, Chinese Academy of Agricultural Sciences, Ministry of Agriculture and Rural Affairs of People's Republic of China, Beijing 100193.

Correspondence and requests for materials should be addressed to H.J. and L.Z. (Phone: +8610-62893622; Email: ptnpc@vip.163.com; Email: Lanzhang@ippcaas.cn)

Supplementary Figure 1. Full-length gels for Figure 4.

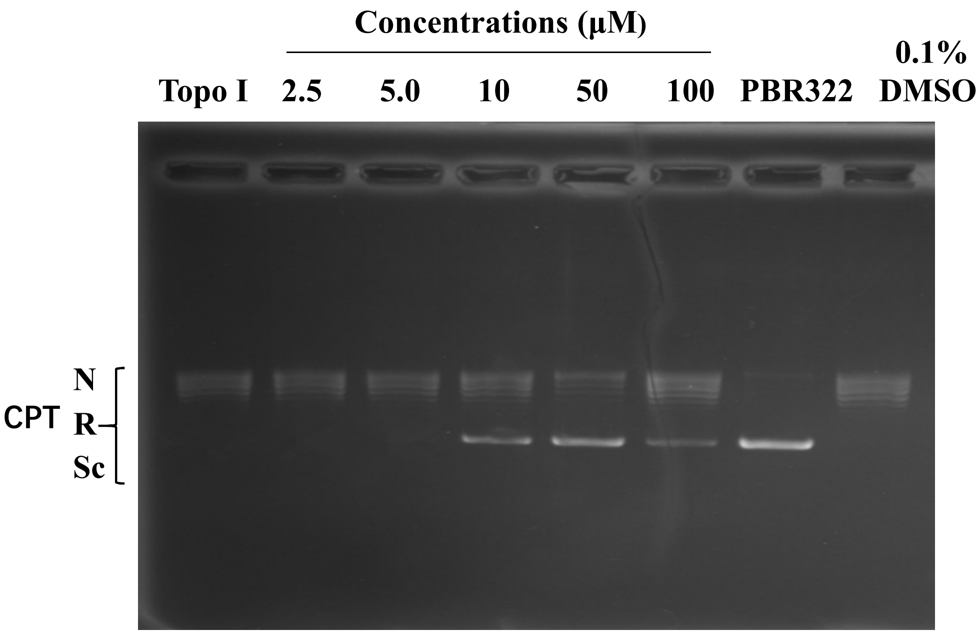

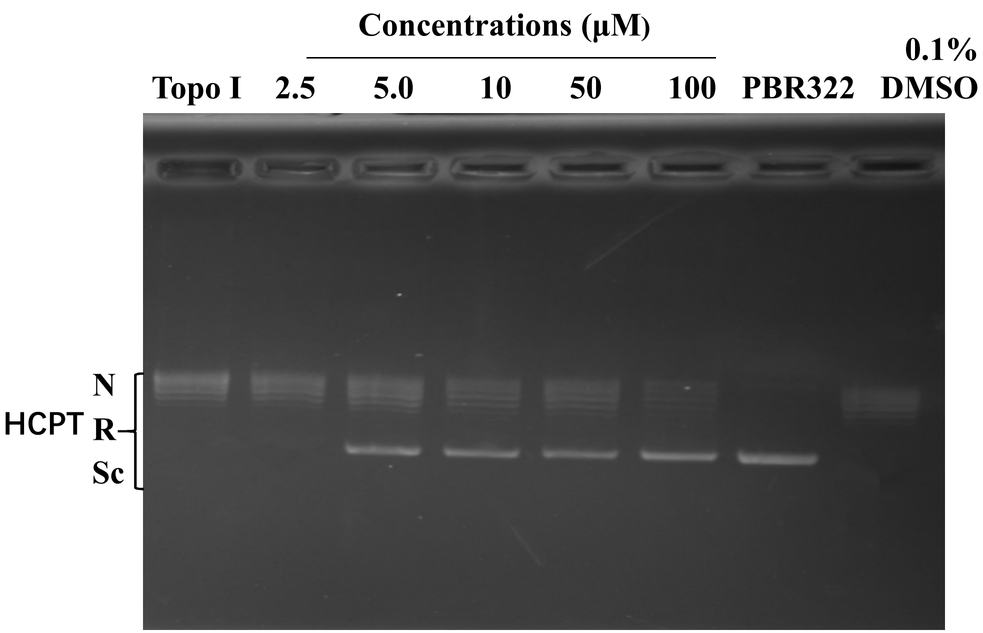

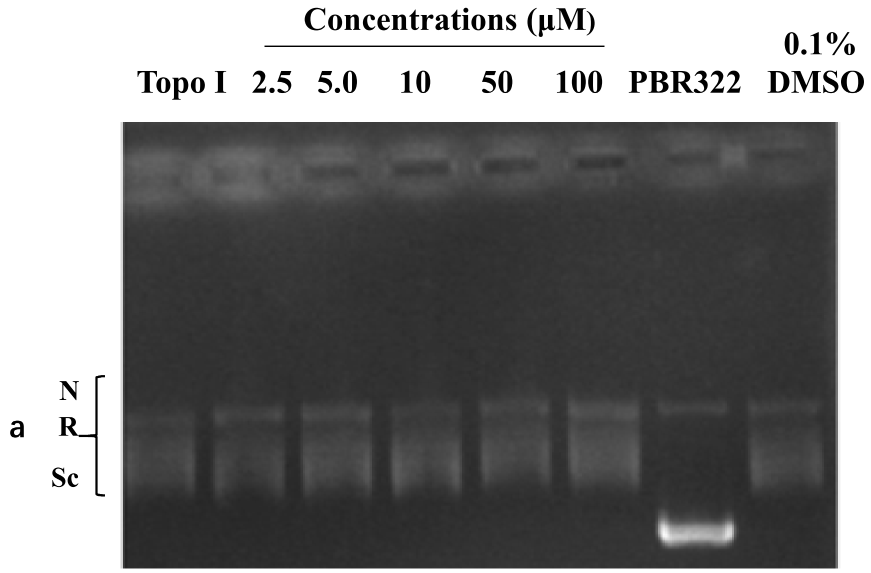
Concentrations (μM)
Topo I
2.5
5.0
10
50
100
PBR322
0.1% DMSO
N
R
Sc
a

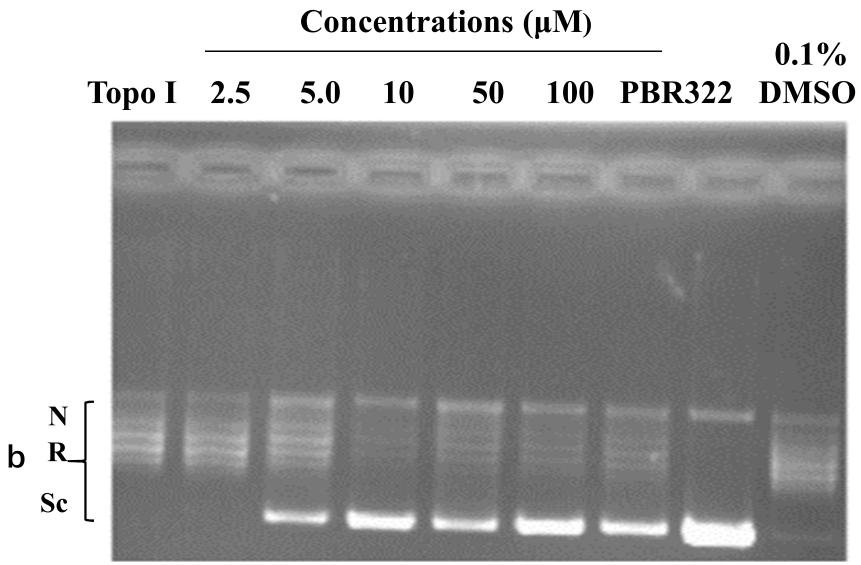
Concentrations (μM)
Topo I
2.5
5.0
10
50
100
PBR322
0.1% DMSO
N
R
Sc
b
